# Low Parasite Load Estimated by qPCR in a Cohort of Children Living in Urban Area Endemic for Visceral Leishmaniasis in Brazil

**DOI:** 10.1371/journal.pntd.0001955

**Published:** 2012-12-13

**Authors:** Letícia Helena dos Santos Marques, Luciana Inácia Gomes, Iara Caixeta Marques da Rocha, Thaís Almeida Marques da Silva, Edward Oliveira, Maria Helena Franco Morais, Ana Rabello, Mariângela Carneiro

**Affiliations:** 1 Laboratório de Epidemiologia de Doenças Infecciosas e Parasitárias, Departamento de Parasitologia, Instituto de Ciências Biológicas, Universidade Federal de Minas Gerais, Belo Horizonte, Brazil; 2 Laboratório de Pesquisas Clínicas, Centro de Pesquisas René Rachou, Fundação Oswaldo Cruz, Belo Horizonte, Brazil; 3 Secretaria Municipal de Saúde, Prefeitura de Belo Horizonte, Belo Horizonte, Brazil; 4 Pós-graduação em Ciências da Saúde: Infectologia e Medicina Tropical, Faculdade de Medicina, Universidade Federal de Minas Gerais, Belo Horizonte, Brazil; Institut Pasteur de Tunis, Tunisia

## Abstract

**Background:**

An important issue associated with the control of visceral leishmaniasis is the need to identify and understand the relevance of asymptomatic infection caused by *Leishmania infantum*. The aim of this study was to follow the course of asymptomatic *L. infantum* infection in children in an area of Brazil where it is endemic. The children were assessed twice during a 12-month period.

**Methodology:**

In this population study, 1875 children, ranging from 6 months to 7 years of age, were assessed. Blood samples were collected on filter papers via finger prick and tested by ELISA (*L. infantum* soluble antigen and rk39). Seropositives samples (n = 317) and a number of seronegatives samples (n = 242) were subjected to qPCR. After 12 months, blood samples were collected from a subgroup of 199 children and tested for *Leishmania* spp. to follow the course of infection.

**Principal Findings:**

At baseline qPCR testing identified 82 positive samples. The prevalence rate, as estimated for 1875 children based on the qPCR results, was 13.9%. The qPCR testing of whole blood samples collected from a cohort of children after 12 months (n = 199) yielded the following results: of the 44 (22.1%) children with positive qPCR results at baseline, only 10 (5.0%) remained positive, and 34 (17.1%) became negative; and of the 155 (77.9%) children with negative qPCR results, 131 (65.8%) remained negative, and 24 (12.1%) became positive at the follow-up measurement. The samples with positive findings at baseline (n = 82) had a mean of 56.5 parasites/mL of blood; and at follow-up the mean positive result was 7.8 parasites/mL.

**Conclusions:**

The peripheral blood of asymptomatic children had a low and fluctuating quantity of *Leishmania* DNA and a significant decrease in parasitemia at 1-year follow-up. Quantitative PCR enables adequate monitoring of *Leishmania* infection.

## Author Summary

In Brazil, visceral leishmaniasis (VL) is caused by the parasite protozoon *Leishmania* (*Leishmania*) *infantum* (syn *chagasi*), which is transmitted to humans by sandflies that have widespread wild and domestic animal reservoirs. An important issue associated with the control of VL is the need to identify and understand the relevance of asymptomatic infection in the transmission cycle of the parasite. We evaluated the course of asymptomatic infection in children by using molecular methods to detect parasite DNA and to monitor parasitemia level over time. The prevalence of VL, as detected by means of a molecular method, for 1875 children was 13.9%. The data demonstrated a significant decrease in parasitemia after 1 year. Furthermore, the level of parasitemia was generally lower in asymptomatic children than in sick children. None of the asymptomatic children showed signs or clinical symptoms associated with VL throughout the duration of the study. This molecular method of measuring parasitemia is adequate for monitoring VL transmission in areas where it is endemic.

## Introduction

Visceral leishmaniasis (VL) is a serious public health problem worldwide, and approximately 500,000 new cases are reported each year [1]. Brazil accounts for 90% of cases in the Americas, where the causative agent of this endemic disease is *Leishmania infantum*, an intracellular protozoan of the *Leishmania donovani* complex [2]. VL in Brazil has long been a typical rural zoonosis. However, since 1980, this disease became a serious and emerging public health problem in the expanding urban centers of several Brazilian cities with different patterns of economic and social development [3]. In Brazil, 3526 new cases of visceral leishmaniasis were reported in 2010. Of these cases, 13.3% were confirmed in the state of Minas Gerais [4]. In 2011, in accordance with the Municipality Health Service of Belo Horizonte, state capital of Minas Gerais, the incidence rate of VL was 6/100,000, and the fatality rate was 13.9%. Asymptomatic carriers are often reported in studies conducted in VL-endemic areas of Brazil, and they are usually more frequent than clinical cases [5]–[7]. Identifying and understanding the relevance of asymptomatic infection during the parasite transmission cycle is currently one of the key issues associated with the control of visceral leishmaniasis [2].

Difficulty in diagnosing asymptomatic VL because of low levels of antibodies and parasites has been reported [8], [9]. Currently, molecular techniques such as polymerase chain reaction (PCR) are used to confirm the diagnosis of VL. These techniques have good sensitivity (75%–98%) and excellent specificity (97%–100%). These methods are also more accurate than serological techniques in identifying infection in asymptomatic carriers [8]–[11]. Real-time quantitative PCR (qPCR) is a variation of conventional PCR and allows for not only the detection of parasite DNA, but also the accurate quantification of copies of the target DNA sequence.

The aim of this study was to follow the course of asymptomatic *L. infantum* infection in children living in disease-endemic areas of the city of Belo Horizonte, Minas Gerais, Brazil, by using qPCR to detect parasite DNA as well as to monitor parasitemia over time.

## Materials and Methods

### Ethical considerations

This study was approved by the research ethics committees of the Universidade Federal de Minas Gerais (No 253/09), Belo Horizonte City Hall (080.2008), and the Centro de Pesquisas René Rachou (01/2010). Legal guardians of the children involved in this study were required to sign the Informed Consent Form at baseline and follow-up phases. Furthermore, the children were granted medical care and treatment when necessary.

### Study design and population

A population-based survey, followed by a cohort study, was carried out from 2009 to 2010. The purpose of this study was to identify and monitor asymptomatic infection of *L. infantum* in children under the age of 7 years living in Belo Horizonte, the capital of Minas Gerais, using qPCR. Belo Horizonte is located 859.19 meters above sea level and between latitudes 44°03′47″and 43°51′27″ and longitudes 19°46′35″ and 20°03′34″. It has a population of 2,375,244 inhabitants [12]. The regional climate is predominantly tropical, with rainy summers and dry winters. This study was conducted in three contiguous geographic subareas in the northwestern region of Belo Horizonte. These subareas were chosen because they have different histories of disease progression and control.

The necessary sample size of approximately 1875 children was estimated according to the following parameters: prevalence of asymptomatic infection rate ranged from 2.4% to 5.6%, based on a study conducted in the metropolitan region of Belo Horizonte [13]; α = 0.05; 1−β = 0.80; and an estimated precision of 0.08.

Initially, the children living in the study area were randomly selected based on the municipal census, which is conducted through the Family Health Program, a strategy currently adopted by the Brazilian Unified Health System (Sistema Único de Saúde - SUS]. These data are periodically updated by community health workers who conduct a census in the areas studied.

Serological tests were performed to identify asymptomatic carriers, followed by qPCR for VL diagnosis and estimation of parasitemia in the sample group. After 12 months of follow-up, a subgroup of the survey sample was assessed to determine the course of infection. Blood samples were collected from these patients for further molecular, hematological, and biochemical tests. Further clinical evaluations were conducted by medical specialists at the health centers in the study regions to detect the presence or absence of signs and symptoms of VL.

### Sample collection

The collection of data and biological material at baseline (survey) was performed at the children's homes by trained staff. Finger-prick blood samples were collected on filter paper (Whatman, number 4). Data regarding the children were recorded on standardized forms. The samples were dried and stored at −20°C until testing. Twelve months after the initial survey, samples were collected at the health centers, the children were clinically examined, and the results were recorded on standardized forms. For this follow-up assessment, peripheral blood samples were collected in appropriate tubes, with ethylene diamine tetra-acetic acid (EDTA).

### Serological tests

Enzyme-linked immunosorbent assays using *L. infantum*–soluble antigen (ELISA-SLA) and recombinant K39 antigen (ELISA-rK39) were performed on blood samples collected on filter paper at baseline, according to the protocol proposed by Pedras *et al*. (2008) and Ho *et al*. (1983) [14], [15].

### Real-time qPCR for detection and quantification of *Leishmania* spp

At baseline, samples that had positive results for *Leishmania* on at least one serological test were also tested by real-time qPCR. In addition, a subgroup consisting of 15% of the serologically negative samples was also evaluated by molecular assay. At follow-up, all whole-blood samples collected with EDTA were tested by qPCR.

The DNA from blood sample collected at both time points was extracted using the DNA extraction kit QIAamp DNA Mini (QIAGEN GMbH; Hilden, Germany), according to the manufacturer's instructions. Three circular fragments, each approximately 5 mm in diameter, were punched from each filter paper blood sample with paper perforators. The protocol for disinfecting the paper perforators that was proposed by Bonne *et al*. [16] was followed to avoid contamination among the samples. The extraction kit was used at follow-up for 120 µL of whole blood, obviously without the step of filter paper elution. The volume of blood subjected to extraction (120 µL) was equivalent to the volume of the eluate obtained from the filter paper used at baseline.

The concentration and purity of the DNA extracted at both time points were determined by spectrophotometry. *A_260_* and *A_280_* were measured with the NanoDrop ND-1000 spectrophotometer (Thermo Fisher Scientific; Wilmington, DE, USA).

The ribosomal RNA small subunit gene (SSU rRNA) is conserved in all species of *Leishmania* and was chosen as the target of qPCR amplification [17], [18]. The forward primer is LEIS.U1 (5′-AAGTGCTTTCCCATCGCAACT-3′), and the reverse primer is LEIS.L1 (5′-GACGCACTAAACCCCTCCAA-3′). These two primers amplify a 67–base pair (bp) fragment of SSU rRNA and were used together with a TaqMan probe, LEIS.P1 (FAM 5′-CGGTTCGGTGTGTGGCGCC-3′ TAMRA) [19]. The primers used in the qPCR protocol in this study are not specific to *L. infantum* and can amplify other organisms of the *Leishmania* genus.

Standard curves were generated for each assay using known amounts of PCR 4 TOPO vector (Invitrogen; São Paulo, Brazil) containing the 67-bp fragment of *L. infantum* SSU rRNA. Serial dilutions (×10) of the recombinant plasmid were made. It is generally accepted that 160 copies of the SSU rRNA gene are present within a single cell of the parasite [17].

Each sample was tested in duplicate in a final volume of 20 µL, including 3 µL of extracted DNA. The final concentrations of the reagents used were as follows: TaqMan MasterMix (Applied Biosystems; Foster, USA) 1×; Probe LEIS.P1, 0.25 pmol/µL; primer LEIS.L1, 0.3 pmol/µL; and primer LEIS.U1, 0.3 pmol/µL. The conditions used for amplification were as follows: 50°C for 2 min for activation of the UDG enzyme, and 95°C for 10 min followed by 40 cycles of 95°C for 15 s for denaturation and 60°C for 1 min for annealing. Values for the threshold of detection and the baseline were automatically determined using StepOne software, v2.1 (Applied Biosystems). A blank consisting of the reaction mixture and water instead of DNA template was added in each qPCR run.

Furthermore, as a control for DNA extraction, amplification, and quality, real-time qPCR assays of the human gene *ACTB* were performed. The primers Aco1 and Aco2 [20], which generate 120-bp fragments, were used in this assay. The cycling parameters were universal, and the melting analysis was conducted based on the parameters of the StepOnePlus Thermal Cycler (Applied Biosystems). SYBR Green (Applied Biosystems) was used for detection of the product.

Samples from five children who received a diagnosis of the clinical form of VL before the treatment were used as positive controls for quantification. DNA was extracted from these samples with the same method used for extraction from whole-blood samples of study participants. The standard curve and the conditions of the qPCR assay were the same.

The parasite load was expressed as the number of parasites present in 1 mL of blood, normalized per nanogram of extracted DNA. This approach neutralizes small variations in the quantification method. Calibrated pipettes were used to test the blood samples collected on filter paper, and the volume of blood in each paper circle was estimated. The estimated volume for the three fragments was approximately 100 µL of blood.

For reproducibility analysis, 10% of the samples collected at both study time points were re-evaluated under the same conditions as those used in the first analysis.

### PCR for *L. infantum*

A PCR assay was performed to confirm infection by *L. infantum*. The chosen primers were sense RV1-CTTTTCTGGTCCCGCGGGTAGG and antisense RV2-CCACCTGGCCTATTTTACACCA, which amplify a fragment with 145 bp of kDNA from *L. infantum*. The protocol for the reaction was proposed by le Fichoux *et al.* (1999) [21].

### Statistical analysis

Databases were generated using EpiData, version 3.2 (EpiData Association; Odense, Denmark) and data were analyzed with STATA 11.0 software (Stata Corp.; College Station, TX, USA).

The qPCR-based prevalence of infection was calculated based on the following parameters: I) the percentage of samples with positive qPCR results and negative serology results; II) the expected number of positive qPCR results if all samples had been tested; and III) the total and estimated numbers of positive qPCR results. Prevalence was assessed as the relationship between the estimated number of positive qPCR results and total number of samples (n = 1875).

The median values of parasite load were calculated and compared by Kruskal-Wallis test at both time points. The agreement between qualitative tests was estimated by the kappa statistic.

## Results

### Quantitative PCR performance

In preliminary experiments, the performance of the *Leishmania* qPCR assay with the TaqMan detection system was assessed through serial dilutions of the linear plasmid DNA containing the 67-bp fragment, with concentrations ranging from 2.8×10^7^ to 2.8×10^2^ copies (analogous to 1.76×10^5^ to 1.7 *Leishmania* cells).

The mean standard curve calculated from 17 independent experiments was linear over at least 6 log_10_ range of DNA concentrations, with a correlation coefficient of 0.989. The PCR efficiency of amplification was 97.4%. The inter-assay coefficients of variation, calculated from triplicate of the 10-fold plasmid DNA dilutions, ranging from 1.7×10^5^ to 1.7 parasites and performed on separated runs, were as follows: 2.35%, 3.05%, 3.64%, 3.27%, 3.35%, and 3.46%, respectively. The intra-assay coefficients of variation were also calculated from triplicate of the same six different concentrations and performed on the same plate. The results were as follows: 0.31%, 0.15%, 0.50%, 0.39%, 0.62%, and 0.98%, respectively.

*Leishmania* qPCR demonstrated a good detection limit, since samples with 2.8×10^2^ copies of linear plasmid DNA, equivalent to 1.7 *Leishmania* cells tested positive. The detection limit was also assessed through serial dilutions of *L. infantum* DNA in water to obtain the points of the curve spanning from 12.000 to 0.012 pg DNA/µL. To convert the amount of DNA detected into number of parasites, the genome size of a diploid *L. infantum* cell (generally assumed to be 3.2×10^7^ bp, [http://www.sanger.ac.uk] was converted into a mass equivalent, yielding a value of approximately 0.070 pg. As the qPCR assay uses 3 µL of DNA template, 0.036 pg of genomic DNA was detected, representing less than a single parasite cell.

Results of melting curve analysis of the samples tested by qPCR of the *ACTB* gene were satisfactory (mean melting temperature: 81.28°C). Therefore, the specific amplification of this gene was confirmed.

The qPCR reproducibility assessment demonstrated regular concordance (kappa = 0.59; 95% CI 0.36–0.80) after evaluation of 10% of the samples collected at both study time points.

### Diagnosis of *Leishmania* infections

#### Baseline

A total of 1875 children participated in the study, 51.1% of whom were girls. The children had a mean age of 42.1±24.7 months and a median age of 42.3 (interquartile range 26–59.4) months, with 7.0% between 2 and 23 months, 33.4% between 24 and 48 months, and 59.6% more than 49 months of age. No signs or symptoms of VL were reported by the guardians of the children at baseline.

The serological assay results showed that of the 1875 children assessed at baseline, 52 (2.8%; 95% CI 2.1–3.6) tested positive by ELISA-SLA, and 280 (14.9%; 95% IC 13.4–16.6) tested positive by ELISA-rk39, totaling 317 children with positive results by at least one serological technique. The overall prevalence rate was 16.9% (95% CI 15.3–18.7).

The first set of qPCR assays was carried out on samples from 559 children from the survey sample. Of these samples, 317 tested positive with at least one serological test and 242 tested negative with all serological tests. These samples with negative results were randomly selected.

Quantitative PCR identified 82 infected samples. Of these, 49 (59.8%) had positive and 33 (40.2%) had negative serology findings. The positivity rate for *Leishmania* spp. in the children studied, as determined by qPCR, was 14.7%. In the group of samples with negative qPCR findings, 209 (37.5%) also tested negative with serology, and 268 (47.9%) tested positive with serology. The results of all tests conducted on the sample group (n = 559) selected at baseline are presented in Table 1. The concordance between serological testing and qPCR was weak (kappa<0.1).

**Table 1:** Agreement between serological and qPCR results for *L. infantum* infection in children (n = 559) living in the northwestern region of Belo Horizonte in 2010.

	qPCR	
Serology	Positive (%)	Negative (%)
Positive, n (%)	49 (8.7)	268 (47.9)
Negative, n (%)	33 (5.9)	209 (37.5)
Total	82 (14.7)	477 (85.3)

Kappa<0.1.

Abbreviation: qPCR, quantitative polymerase chain reaction.

The prevalence estimated by qPCR rate was 13.9% (95% CI 12.4–15.5), according to the parameters described in Table 2 and taking the 1875 children into account.

**Table 2:** Estimate by qPCR of the prevalence of *L. infantum* infection in children living in the northwestern region of Belo Horizonte (n = 1875).

	Positive by qPCR and among negative by serology	Expected number of samples with positive qPCR results	Prevalence (95% CI)
**qPCR**	13.6% (33/242)	260.9 [(1558×0.136)+49]	13.9% (12.4–15.5) (260.9/1875)

Abbreviations: CI, confidence interval; qPCR, quantitative polymerase chain reaction.

#### Follow-up

After 12 months, 199 of the 317 children who had positive by serology and/or by qPCR findings were reevaluated. A total of 118 (37.2%) of the children who tested positive at baseline were lost to follow-up (they could not be found).

The qPCR results of whole-blood samples collected from 199 children 12 months after the initial testing were as follows: of the 44 (22.1%) children with positive qPCR results at baseline, only 10 (5.2%) remained positive, and 34 (17.1) became negative; and of the 155 (77.9%) children with negative qPCR results, 131 (65.8%) remained negative, and 24 (12.1%) became positive during the at follow-up (Table 3).

**Table 3:** Diagnosis of asymptomatic infection in 199 subjects by qPCR at baseline and 12-month follow-up in Belo Horizonte, Minas Gerais (2009–2010).

	Baseline	12-Month Follow-up		
	Positive	Remained positive	Became positive	Total number of positives
qPCR	44 (22.1%)	10 (5.2%)	24 (12.1%)	34 (17.1%)

	Negative	Remained negative	Positive Became	Total Number of Negative
qPCR	155 (77.9%)	131 (65.8%)	34 (17.1%)	165 (82.9%)

Abbreviations: follow-up; qPCR, quantitative polymerase chain reaction.

Electrophoresis from conventional PCR performed on samples with positive qPCR results revealed bands with 145 bp, confirming that the amplified DNA was in fact from *L. infantum*. Thus, we can confirm that the asymptomatic infection diagnosed in this study group was caused by this species.

Values of hemoglobin, leukocytes, and platelets were similar among children with different courses of *Leishmania* DNA results (remained positive, remained negative, became positive and became negative, with no significant statistical difference in any comparison by Kruskal Wallis test, p<0.05). No signs or symptoms suggestive of VL were observed during clinical examination at baseline or follow-up.

### Quantification

**Figure 1:**
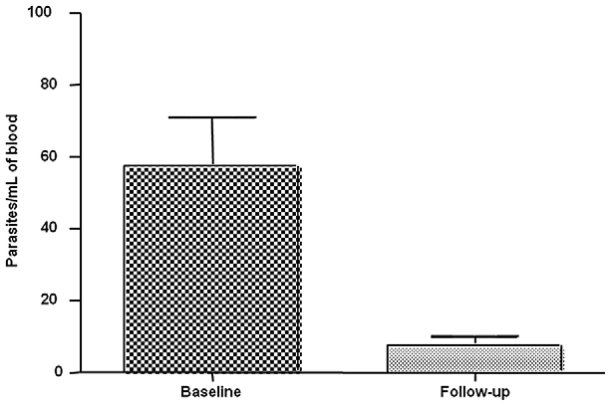
Parasite load of children remained positive tested by qPCR results at baseline and follow-up. The samples that tested positive at baseline had a mean concentration of 56.5±33.4 parasites/mL of blood. At follow-up, the mean parasitemia of children who remained positive was 7.8±7.0 parasites/mL (baseline, dark; follow-up, clear).

Parasite load was assessed at both time points. The samples with positive results at baseline (n = 82) had a mean concentration of 56.5 (±33.4) parasites/mL of blood and a median of 60.9 (range: 16.0–88.8) parasites/mL. At 12-month follow-up, the mean and median parasitemia of the children who remained positive were 7.8 (±7.0) and 5.8 (range: 3.8–6.7) parasites/mL of blood, respectively (Figure 1). The mean and median values for the 24 children who became positive at follow-up were 7.9 (±6.5) and 5.0 (range: 3.3–10.0) parasites/mL of blood, respectively. The Kruskal-Wallis test showed a statistically significant difference between baseline and follow-up values of parasitemia (p = 0.0019*)*.

Children with the clinically manifest form of VL, the positive control group, presented an estimated mean parasitemia of 2190.2 (±694.6) parasites/mL.

## Discussion

The results of this study showed a prevalence of *Leishmania infantum* circulating in 13.9% (95% IC 12.4–15.5) of asymptomatic children younger than 7 years living in an urban area that is endemic for VL. There was a significant decrease in parasitemia in these children after one year. A number of children had varying parasitemia values, but on average, their parasite levels were lower than those of the sick children in our control group.

The implementation of effective measures to control VL is increasingly urgent because of the spread of this endemic disease in urban areas. Diagnostic tests in dogs and humans are essential for identifying the areas with the highest transmission rates. Although dogs are the main reservoir of *L. infantum* in urban areas, the prevalence of asymptomatic VL can serve as an indicator of the extent and maintenance of parasite transmission [5], [8], [22], [23]. Therefore, in this study children were selected for the sample group to allow for the identification of relatively recent transmission events in the study area.

The screening method chosen was serology, because it is more useful when testing many samples. However, several studies have already shown that ELISA is not an accurate technique for the diagnosis of asymptomatic infections; rather, it is better suited for identifying individuals who exhibit clinical signs and symptoms of VL. Therefore, when serology testing alone is used to identify asymptomatic infection, a large number of asymptomatic carriers are likely to be disregarded [24], [25]. In light of this fact, molecular methods have been considered an important complement to serological detection of asymptomatic carriers. Thus, this study used a protocol for the detection of *Leishmania* DNA by qPCR in samples of peripheral blood collected on filter paper via finger prick or venipuncture.

The baseline results showed a weak association between serology and qPCR findings. Of the samples with positive qPCR findings, 59.8% were also seropositive, and 40.2% were seronegative. Differences between these techniques have also been reported by other authors and may be due to serological testing in asymptomatic individuals for whom the tests are not accurate [23]–[26]. Furthermore, the techniques are based on different parameters for detecting infection: antibodies or genomic DNA. Variation in mode of transmission, such as simple contact/exposure versus genuine, established infections, may also be a factor. Moreover, results from the serum-reactive samples may include false-positives.

Because this study aimed to identify areas with recent *Leishmania* transmission events, and given that qPCR identifies the presence of parasite DNA, this molecular method can be considered a technique that provides more robust results than other methods. A recent study demonstrated that *Leishmania* DNA rapidly degrades soon after death of the amastigote. This suggests that results obtained by qPCR are due to parasites that are alive and intact [27].

Standardization of blood extraction from the filter paper allowed us to use qPCR in an epidemiological survey involving a large number of participants. The methods used were adequate and maintained the quality of qPCR quantification. Blood collection on filter paper is an acceptable alternative to venipuncture, especially when the participants are children, because finger pricking is more practical and less invasive. Follow-up blood collection was carried out by venipuncture because of the smaller number of participants, who had previous positive results, and was performed at health centers. Because the children participating in the study live in VL endemic areas where transmission occurs continuously, their infection status may have changed during the follow-up period [28], [29]. Recent infection may be responsible for the positive test results of children who had previously tested negative. In contrast, the 10 children who tested positive at baseline and follow-up may have maintained the original infection or been reinfected.

None of the cohort subjects showed signs or clinical symptoms associated with VL throughout the duration of the study. The absence of clinical signs or symptoms of VL has already been observed in other studies of individuals with *L. infantum* infection, which was detected by molecular tests and evaluated during an extended follow-up period [5], [11], [24].

Quantitative PCR allowed for not only diagnosis, but also quantification of *Leishmania* DNA. Assessment of parasitemia at baseline and follow-up demonstrated a decrease in mean parasite load after 12 months (from 56.5 to 7.8 parasites/mL). This finding suggests that, with regard to the samples that tested positive both times (n = 10), asymptomatic infection may be self-regulated, with the partial or total clearance of parasites, as demonstrated by the patients who tested positive at baseline and negative at follow-up (n = 24).

Possible limitations of this study involve subjects lost to follow-up and the use of different procedures to collect biological material at baseline and follow-up. The main reasons for failure to present for follow-up examination were migration to other areas, denial, and lack of interest in continued participation. A comparison of demographic characteristics, such as age, sex, and duration of residence in the area, revealed no significant differences between those who were and were not included at follow-up. Therefore, selection bias may be ruled out when analyzing the results.

It is important to highlight that despite the use of two different methodologies to collect biological material in both phases of the study, all procedures were performed in such a way as to minimize the possible variations intrinsic to the different techniques: the blood volume used for DNA extraction at follow-up was adjusted to be similar to the volume collected from the filter paper (at baseline), which was approximately 120 µL. Furthermore, the parasite load values calculated at the end of experiments were standardized and normalized per nanogram of extracted DNA, which also minimized potential variations.

The low number of circulating parasites in asymptomatic carriers may influence the reproducibility of the analytic technique (kappa = 0.60) because of the decreased probability of finding parasites in peripheral blood samples. To investigate parasite load, we performed a similar assay, following the same criteria as those used in tests previously performed on samples from symptomatic children with confirmed clinical VL (positive controls). Parasite load was significantly greater in the positive controls than in asymptomatic carriers, with thousands of parasites per milliliter of blood. This finding suggests that parasite load data should not be used alone, but rather in combination with monitoring of each patient or in a comparative manner between groups of individuals with clinical presentation of confirmed VL and asymptomatic individuals. Recent studies showed that the level of parasitemia estimated by qPCR is related to clinical patient profile [30], [31].

Molecular techniques, especially qPCR, to estimate the prevalence and incidence of infection can be ideal for epidemiological studies because they can detect and quantify *Leishmania* DNA, which is a reliable marker of infection [32]. However, the greater complexity and cost involved in performing qPCR on a large number of samples, compared with the complexity and cost associated with immunological tests, must be taken into account.

In conclusion, the findings of this study demonstrate the usefulness of qPCR for epidemiological studies monitoring parasitemia in asymptomatic individuals. Despite the cost, qPCR performance was satisfactory. It detected the clearance of parasite DNA, which may be relevant in cases of infection in which the tests are significant limitations on the interpretation of their results. Therefore, this method allows for better monitoring of *Leishmania* infection in areas where it is endemic.

## Supporting Information

Checklist S1 **STROBE checklist.**
(DOC)
